# Fire-stimulated flowering enhances multiple plant fitness components

**DOI:** 10.1093/aob/mcag048

**Published:** 2026-03-16

**Authors:** Julia Gegunde, Maria Clara Castellanos, Juli G Pausas

**Affiliations:** CIDE (CSIC, UV, GV), Centro de Investigaciones sobre Desertificación, Montcada, Valencia 46113, Spain; Department of Ecology & Evolution, School of Life Sciences, University of Sussex, Brighton BN1 9QG, UK; CIDE (CSIC, UV, GV), Centro de Investigaciones sobre Desertificación, Montcada, Valencia 46113, Spain

**Keywords:** Adaptive trait, Asparagales, fire ecology, Mediterranean geophytes, pollination, post-fire flowering, post-fire recruitment

## Abstract

**Background and Aims:**

Fire is a key ecological driver shaping reproductive strategies in fire-prone ecosystems. One distinctive strategy is fire-stimulated flowering, whereby plants flower more profusely under post-fire conditions than in the absence of fire. Despite its widespread occurrence, the reproductive benefits of this strategy remain poorly quantified. We hypothesized that fire-stimulated flowering is an adaptive strategy that enhances plant fitness by increasing success across multiple reproductive stages.

**Methods:**

We compared multi-year data on all stages of reproduction, from flowering density to seedling recruitment, in four Mediterranean geophytes (*Asphodelus cerasifer*, *Dipcadi serotinum*, *Drimia maritima* and *Narcissus assoanus*), between burned and adjacent unburned areas across six natural wildfires.

**Key Results:**

Plants in burned areas showed higher flowering density, flower production and total seed output, and in some species, this was also associated with increased pollinator visitation and pollen deposition. Seedling recruitment was consistently higher in burned areas.

**Conclusions:**

Our results provide evidence of the adaptive value of fire-stimulated flowering, which allows plants to exploit the unique abiotic and biotic conditions of the immediate post-fire environment, and highlight fire as a key driver of plant life-history evolution in Mediterranean ecosystems.

## INTRODUCTION

Fire is a fundamental ecological and evolutionary process in many ecosystems worldwide. Plants living in fire-prone ecosystems have acquired a range of strategies to survive and reproduce after fire (Keeley *et al*., 2011; McLauchlan *et al*., 2020; Keeley and Pausas, 2022). One is to quickly and profusely flower post-fire, a strategy known as fire-stimulated flowering (Lamont and Downes, 2011; Pyke, 2017). This strategy occurs across a diverse range of plant growth forms (shrubs, subshurbs, trees, palm-like plants and herbaceous species), but is particularly common among deciduous geophytes (Lamont and Downes, 2011) as their buds are well-insulated from the heat of fires beneath the soil (García and Zamora, 2003). Their below-ground organs, such as bulbs, corms, rhizomes or tuberous roots, support rapid flowering after fire even among individuals with dormancy periods maintained for several reproductive cycles (Dafni *et al*., 1981*a*; Le Maitre and Brown, 1992).

Fire-stimulated flowering is thought to confer reproductive benefits to species living in fire-prone ecosystems (Lamont and Downes, 2011; Pyke, 2017). These plants typically flower within a few weeks or months after the fire, even days in some cases (Fidelis *et al*., 2019; Pilon *et al*., 2023), and reach a blooming peak shortly thereafter. Therefore, the window of opportunity for maximizing reproductive success is expected to align with the immediate post-fire period (less than a year), when species take advantage of reduced competition and higher availability of light and nutrients post-fire (Caon *et al*., 2014; Tosatto *et al*., 2025). Many previous studies have examined this process several years after a fire (Ne’eman *et al*., 2000; Potts *et al*., 2006) rather than during the critical post-fire period, underestimating the reproductive benefits associated with fire-stimulated flowering. Furthermore, many other studies rely on prescribed fires as experimental tools to assess the effects of fire on flowering and reproductive output (Vickery, 2002; Borchert and Tyler, 2009; Wagenius *et al*., 2020; Richardson *et al*., 2023). Prescribed fires are typically conducted under controlled conditions, which often result in lower fire intensity, limited spatial extent and atypical seasonal timing, potentially leading to an underestimation of the reproductive benefits of fire-stimulated flowering.

The environment that plants experience immediately after a fire is notably different from the pre-fire conditions, including their pollination environment. In species with fire-stimulated flowering, fire synchronizes flowering, both spatially and temporally, increasing the density of flowering individuals (Wagenius *et al*., 2020; Richardson and Wagenius, 2022). Additionally, the higher availability of abiotic resources may promote a greater number of flowers per plant, further contributing to the increased availability of flowers for pollinators in the population (Vickery, 2002; Borchert and Tyler, 2009). Many studies have also explored the effect of fire on direct changes in the pollinator community (Carbone *et al*., 2019). Fire-sensitive pollinator species may initially experience a reduction in abundance, but this is not always the case, and post-fire conditions provide abundant nesting resources, such as increased bare ground, dead wood and nesting cavities (Potts *et al*., 2005; Cane and Neff, 2011; Mola and Williams, 2018; Simanonok and Burkle, 2019), promoting the rapid recolonization of pollinators from unburned areas. Recolonization processes may ultimately contribute to an increase in pollinator abundance in burned areas, a process that depends on the spatial arrangement of burned and unburned habitats within pollinator foraging ranges (Ulyshen *et al*., 2024). Thus, the spatial scale of the post-fire landscape may also influence pollination processes (Brown *et al*., 2016).

An increased flower availability and greater number of pollinators visiting flowers after fire may potentially lead to a higher proportion of pollinated flowers and increased pollen load on the stigmas (pollination quantity). In terms of pollination quality, immediately after a fire, the floral community will have fewer co-flowering species, which is expected to reduce sharing of pollinators (Mitchell *et al*., 2009; Albor *et al*., 2024) and potentially increase the quality of pollen received (Ashman and Arceo-Gómez, 2013). Nevertheless, the increase of population density and flower abundance may also increase the risk of inbreeding, either through geitonogamy (i.e. pollination within the same individual) or biparental inbreeding (i.e. mating between genetically related individuals within the population), reducing the pollination quality, especially in self-incompatible species (LoPresti *et al*., 2018; Wagenius *et al*., 2020; Richardson and Wagenius, 2022). Previous research has shown that fires promote floral visitation by pollinators at the plant community level (Carbone *et al*., 2025), but little is known about the implications for the plants at the individual level in terms of flower visitation rates or pollen deposition by pollinators (but see Ne’eman *et al*., 2000; Potts *et al*., 2006).

The effects of fire-stimulated flowering can extend beyond pollination. Previous studies have documented an increase in absolute number of fruits and seeds per individual plant after fire (Carbone *et al*., 2025) but its consequences for seedling recruitment and the implications for population dynamics are still underexplored (Vickery, 2002; Diadema *et al*., 2007). Fire improves the conditions for seedling recruitment and establishment by increasing light, nutrients and space. Furthermore, there is evidence that fire can reduce antagonistic interactions, such as seed predation and seedling herbivory (Vickery, 2002; García *et al*., 2016). Together, these improved abiotic conditions and lower biotic pressures may create a window of opportunity for seedling recruitment, particularly for fire-stimulated flowering species capable of setting seeds shortly after the fire (Keeley *et al*., 2006; Beck and Wagenius, 2024; Beck *et al*., 2024). Although evidence that species with fire-stimulated flowering can benefit from fire is accumulating (Carbone *et al*., 2025), few studies have demonstrated these benefits on the different stages of the reproductive cycle in the same plant populations (Vickery, 2002; Diadema *et al*., 2007; Richardson *et al*., 2023; Tosatto *et al*., 2025), leading to an incomplete understanding of the strategy. Because multiple ecological processes and demographic bottlenecks operate between flower production and the establishment of an adult individual, it is important to consider multiple stages of the reproductive cycle when assessing fire effects on fire-stimulated flowering. We hypothesized that, in fire prone-ecosystems, fire-stimulated flowering provides fitness benefits under recurrent fires, and that these benefits may occur at different stages of their reproductive cycle, from flowering initiation to seedling recruitment. To test this, we studied four species of geophytes in Mediterranean shrublands recently affected by wildfires. For each species we assessed the effect of fire on flower density and production, pollination, fruit and seed production, and seedling recruitment, for a holistic understanding of the fitness benefits of fire-stimulated flowering.

## MATERIAL AND METHODS

### Study species and design

**Fig. 1.**
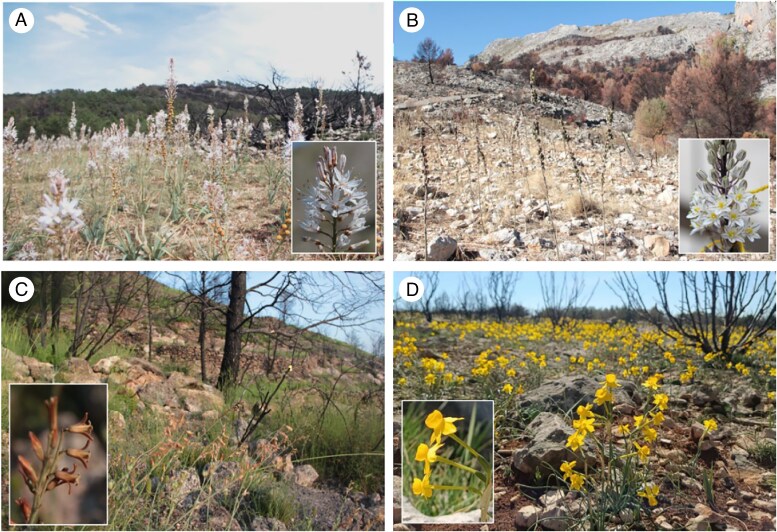
*Asphodelus cerasifer* (A), *Drimia maritima* (B), *Dipcadi serotinum* (C) and *Narcissus assoanus* (D) exhibiting profuse synchronized flowering (already fruiting in the case of *Drimia maritima*) in recently burned areas. Close-up photos in each panel show details of the flowers of each species. Photos by J. Gegunde (A,B,C) and J.G. Pausas (D).

The study was conducted from 2020 to 2023 on the east of the Iberian Peninsula, a region characterized by a typical Mediterranean climate, where summer wildfires are common (Pausas and Paula, 2012). We surveyed recently burned areas during the first few months after wildfires and selected four geophyte species widely distributed in our study region that exhibited fast post-fire flowering: *Asphodelus cerasifer* J.Gay (Asphodelaceae)*, Dipcadi serotinum* (L.) Medik. (Asparagaceae), *Drimia maritima* (L.) Stearn (Asparagaceae) and *Narcissus assoanus* Dufour (Amaryllidaceae) (Fig. 1).

*Asphodelus cerasifer* has a rhizome surrounded by tuberous roots, while the other species possess bulbs. All species produce clonal units (i.e. ramets) that can survive and reproduce sexually and independently; in this study, we refer to these clonal units as *individuals*. Leaves of all species dry out during summer and are typically dry by the time the fire occurs. *Drimia maritima* is hysteranthous (leaves and flowers appear in separate seasons) and flowers at the end of the summer, whereas the other species are synanthous (leaves and flowers co-occur) and flower in the spring.

*Drimia maritima* (Dafni and Dukas, 1986)*, N. assoanus* (Baker *et al*., 2000*b*) and *A. cerasifer* (Lifante and Valdés, 1994; Lifante, 1996) are insect-pollinated. *Asphodelus cerasifer* and *Drimia maritima* are self-compatible (partially, in the case of *Drimia maritima*), although pollination by insects is required for effective pollination owing to the spatial separation of the anthers and stigmas in their large, open flowers (Fig. 1). *Narcissus assoanus* is self-incompatible and presents heterostylous, tubular flowers that limit autonomous selfing (Baker *et al*., 2000*b*). Information on the reproductive biology of *Dipcadi serotinum* is scarce, but related species in the genus *Dipcadi*, with similarly tubular, brownish flowers, are typically self-incompatible and pollinated by nocturnal insects (Manning *et al*., 2012; Rodrigues *et al*., 2024). Consistent with this pattern, a preliminary pollinator exclusion experiment in *Dipcadi serotinum* (*N* = 11) showed no fruits set in bagged plants while most individuals exposed to pollinators produced fruits, indicating that the species does not self-pollinate and relies on pollinators for successful reproduction (see Supplementary Data Table S1). All species exhibit short-distance seed dispersal and, to our knowledge, do not form a permanent seed bank. None of the studied species are dispersed by vertebrates; but we cannot rule out dispersal by small invertebrates, such as ants.

### Sampling scheme

The localities selected for the study are Mediterranean shrublands dominated by *Cistus* sp. *pl., Ulex parviflorus*, *Salvia rosmarinus*, *Juniperus oxycedrus*, *Quercus coccifera*, *Chamaerops humilis* and *Brachypodium retusum*. *Asphodelus cerasifer* was studied in three localities, *Drimia maritima* in two localities, and *Dipcadi serotinum* and *N. assoanus* in a single locality (Table 1; Supplementary Data Table S2, Fig. S1 for more details). For each species and locality, we sampled two to four spatially separated sites per fire treatment (recently burned areas and adjacent unburned areas), with the number of sites determined by the local availability of each species (except for *Drimia maritima* in Jubrique, where one site per fire treatment was available; see Table 1 for details on sample sizes). Burned sites were located in the centre of the burned areas to avoid proximity to the edge of the fire, while the unburned sites were carefully chosen to be representative of pre-fire conditions, ensuring similar orientation, altitude and vegetation composition.

**Table 1.** Localities per species, wildfire initiation dates at burned areas, sampling period and details on the sampling effort. The number of sites sampled, plots (for estimates of resprouting and flowering density), pollinator censuses conducted, styles analysed and individuals sampled are reported separately for burned (B) and unburned (U) areas.

Species	Locality	Wildfire initiation	Sampling period	Sites (B, U)	Plots (B, U)	Pollinator censuses (B, U)	Styles (B, U)	Individuals (B, U)
*Asphodelus cerasifer*	Azuébar (Castellón)	Aug 2021	Mar 2022–Jan 2023	2, 4	33–36	97, 32	62, 60	57, 96
	Bejís (Castellón)	Aug 2022	Mar–Jul 2023	2, 3	29–40	75, 79	66, 56	82, 118
	Vall d’Ebo (Alicante)	Aug 2022	Mar–Jul 2023	3, 3	41**–**34	96, 117	60, 53	126, 84
*Dipcadi serotinum*	Azuébar (Castellón)	Aug 2021	Mar 2022–Jan 2023	2, 3	95**–**100	40, 30	19, 19	141, 110
*Narcissus assoanus*	Costur (Castellón)	Aug 2022	Mar–Jul 2023	2, 3	80**–**79	64, 76	58, 54	480, 452
*Drimia maritima*	Beniardà (Alicante)	Jul 2020	Sep–Dec 2020	3, 2	70**–**53	–	–	64, 52
	Jubrique (Málaga)	Sep 2021	Sep–Dec 2021	1, 1	–	–	–	23, 21

#### Resprouting and flowering density

At the end of the flowering season, we estimated plant density for each species and locality in sites located at the centre of the burned areas and in adjacent unburned areas. Additionally, we sampled plant density at the edge of the fire, where a partially burned population included both burned and unburned adjacent areas under the same local environment. For *Drimia maritima*, plant density was quantified in only one of the two sampled localities (Beniardà), and for *A. cerasifer*, plant density at the fire-edge was assessed only in Azuébar (as this species was missing at fire-edges in the other two localities). Plant density was estimated using plots whose size and number were adjusted to the specific characteristics of each species (i.e. plant size and spatial distribution; see Table 1 for details on the number of plots): 20 m^2^ (10 × 2 m) plots for *A. cerasifer* and *Drimia maritima*, and 1 m^2^ (1 × 1 m) plots for *Dipcadi serotinum* and *N. assoanus*. For each plot, we counted the total number of individuals resprouting in the burned areas or sprouting in the unburned areas (hereafter *resprouting individuals*) and the number of individuals flowering. For *Dipcadi serotinum* and *N. assoanus*, very few individuals, if any, resprouted without flowering (and, if so, they were difficult to identify), so we equalled the number of flowering individuals and the number of resprouting individuals.

#### Reproductive output

We conducted timed surveys (hereafter ‘censuses’) of pollinator activity to assess pollinator visitation to *A. cerasifer*, *Dipcadi serotinum* and *N. assoanus* in burned and adjacent unburned sites (see Table 1 for sample sizes). Each census consisted of a 3-min observation of pollinator activity to a floral patch from 0900 to 1400 h in the case of *A. cerasifer* and *N. assoanus* and from 2000 to 2200 h for *Dipcadi serotinum*, as we expected mainly nocturnal pollination for this species. Prior to each census, we counted the total number of open flowers available for pollinators per patch. We recorded the total number of flowers visited by each pollinator during censuses; pollinators were identified later using close-up photographs. We calculated the per-time unit probability that an individual flower was visited (*flower visitation rate*) by considering the number of open flowers that were visited and those that remained unvisited in each flower patch. After the flowers withered, we collected styles in vials with 70 % ethanol (Table 1). In the laboratory, we stained the stigmas with fuchsine and counted the number of conspecific pollen grains (pollen deposition) using a light microscope.

We haphazardly selected at least 20 individuals for each burned and unburned site per species (see Table 1 for sample sizes) to evaluate the total number of flowers and fruits produced, and to estimate the fruit set per individual (proportion of flowers setting fruit). Individuals were spaced sufficiently apart to ensure they were not ramets from the same maternal plant. For *Dipcadi serotinum* we also recorded the number of floral scapes produced per individual and the number of flowers produced per scape (the other species produce only one floral scape per individual). We also recorded the number of seeds per fruit for one to five fruits (depending on the species) per individual and estimated the total seed production for a subset of individuals producing fruits (see Supplementary Data Table S7 for sample sizes).

A few months after seed dispersal, coinciding with the wet season, we estimated seedling recruitment in burned and unburned sites by counting the number of seedlings per plot for the two study species flowering in 2022: using 10 m^2^ (5 × 2 m) plots for sampling *A. cerasifer* (25 plots in burned and 15 in unburned areas), and 1 m^2^ (1 × 1 m) plots for *Dipcadi serotinum* (35 plots in burned and 49 in unburned areas). In 2023, no recruitment was recorded for any species because the spring was too dry for any germination to occur.

### Data analyses

We performed separate linear mixed models to evaluate the effect of fire on each of the different stages of the reproductive cycle that we measured on each species. All analyses included fire treatment as a categorical predictor variable with two levels (burned and unburned). Locality and its interaction with fire were also included in those models with response variables measured across more than one locality (for *A. cerasiferus* and *Drimia maritima*), and site as a random factor in all cases. See Supplementary Data Table S3 for a summary of the fitted model structures.

#### Resprouting and flowering density

To analyse the effect of fire (or the effect of locality and its interaction with fire when applicable) on the number of individuals resprouting per plot (resprouting density) and the number of individuals flowering per plot (flowering density), we fitted separate generalized mixed models (GLMMs) with a Poisson error distribution. To test the effect of fire on the proportion of individuals resprouting that were also flowering, for *A. cerasifer* and *Drimia maritima* (for *Dipcadi serotinum* and *N. assoanus* all the individuals resprouting were also flowering), we fitted GLMMs with a binomial error distribution. For each response variable and species, we performed separate models using (1) data only from the sites in the centre of the burned areas and adjacent unburned areas and (2) data only from the partially burned population at the edge of the fire. The latter approach allows us to assess the impact of fire while controlling for between-site variation in population density, as the burned and unburned individuals at the fire edge belong to the same population. We included plot as an additional random effect (an observation-level random effect) when needed to deal with overdispersion (Bolker, 2015).

#### Reproductive output

Number of flowers per individual (log), seeds per fruit (square-root) and total seed production per individual (square-root) were transformed and used as response variables in linear mixed models (LMMs).

Flower visitation rate was included as the response variable in GLMMs with a binomial error distribution, including an observation-level random effect when necessary to account for overdispersion, as above (Bolker, 2015). The number of pollen grains counted on the stigma (pollen deposition) was included as the response variable in a GLMM with a negative binomial error distribution for *A. cerasifer*, and in LMMs for *N. assoanus* and *Dipcadi serotinum* (data for the latter were log-transformed to achieve normality).

For percentage of flowers setting fruits (fruit set) we fitted a GLMM with a binomial error distribution while for the number of seedlings per plot we used a GLMM with a negative binomial error distribution. We included plant identity as an additional random effect in the models for seeds per fruit and fruit set.

The focus of the paper is to test the effect of fire (qualitative factor); however, our sampling sites inevitably have some variability in the distance to the nearest unburned area (i.e. the edge of the fire). To evaluate its potential effect, we added distance to the edge of the fire as a covariable in the models related to pollination: flower visitation rate, pollen deposition, fruit set, seeds per fruit and total seed production. The distance to the edge for unburned sites was included as 0. This variable was finally included in the models when significant.

We also evaluated the effect of fire across species on each stage of the reproductive cycle by comparing the mean and the standard deviation of each variable, location and species in burned and unburned conditions. Because response variables varied in scale and variability among species, we used the unbiased standardized mean difference (Hedges’ *g*). Effect sizes were then analysed using multilevel mixed-effects meta-analytical models, including response variable as a moderator, and species and locality as nested random effects to account for non-independence among effect sizes (Gurevitch and Hedges, 2001). This approach allowed fire effects to be estimated across species and directly compared among variables. We also estimated the overall mean (pooled) Hedges’ *g* across all response variables to quantify the general effect of fire. A positive effect size indicates an increase in the reproductive variable after fire relative to the unburned condition, whereas a negative effect size indicates the opposite.

All statistical analyses were performed in the R software v.4.1.1 (R Core Team, 2024). GLMMs and LMMs were performed with the ‘lme4’ package (Bates *et al*., 2015). When the fire × locality interaction term was significant, we conducted pairwise comparisons (*post hoc* analysis) of the estimated marginal means with the ‘emmeans’ package (Lenth, 2024). We checked uniformity of residuals and overdispersion using the ‘DHARMa’ package (Hartig, 2022). Effect sizes and model fit were computed using the ‘metafor’ package (Viechtbauer, 2010).

## RESULTS

### Resprouting and flowering density

**Fig. 2.**
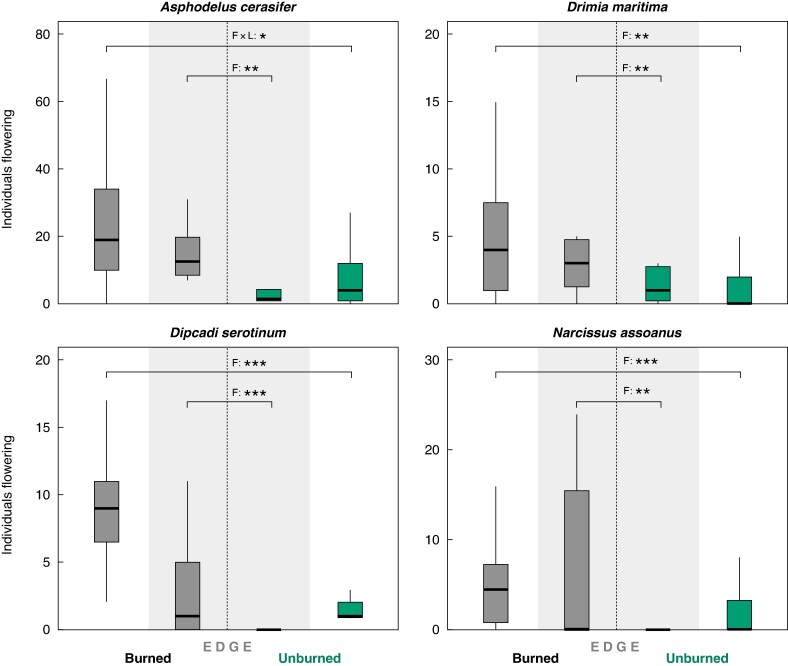
Number of individuals flowering per plot (flowering density) in the unburned (in green) and burned (in grey) areas. The shaded portion of each graph shows data from the partially burned populations on the edge of burned areas, while non-shaded parts of the figure show data from the sites in the centre of the burned areas and adjacent unburned areas. Boxplot boundaries indicate the 25th and 75th percentiles, the horizontal line inside the box marks the median and whiskers extend up to 1.5 times the interquartile range. Asterisks indicate the significance level of fire (F), locality (L) and their interaction (F × L) for *Asphodelus cerasifer* and *Drimia maritima*; and fire (F) only for *Dipcadi serotinum* and *Narcissus assoanus*: *P* < 0.05 (*), *P* < 0.01 (**), *P* < 0.001 (***); non-significant comparisons are not marked. See Supplementary Data Table S4 for summary statistics.

**Fig. 3.**
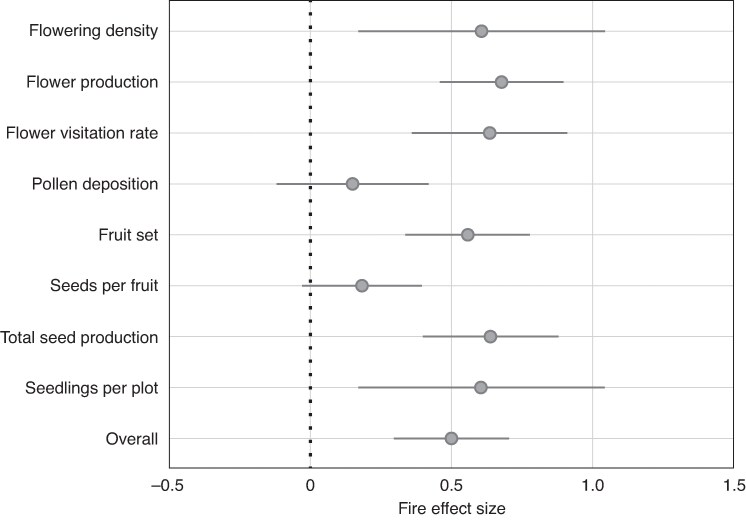
Fire effect sizes (mean Hedges’ *g*) for each stage of the reproductive cycle and across stages (overall) when analysing all species combined. Points represent model-estimated mean Hedges’ *g* from multilevel meta-analytical models, and horizontal lines indicate 95 % confidence intervals. Positive values indicate higher responses in burned areas, whereas negative values indicate higher responses in unburned areas. Effects were considered statistically significant when confidence intervals did not include zero. See Supplementary Data Table S5 for summary statistics.

Fire positively affected the density of flowering individuals both when species were analysed separately (Fig. 2; Supplementary Data Table S4) and combined (see Fig. 3; Table S5). Flowering density (number of individuals flowering per plot) increased significantly for each species in the burned areas, whether analysing data from (1) the centre of the burned and adjacent unburned areas or from (2) the partially burned populations at the fire-edge. For *A. cerasifer*, the fire × locality interaction was significant when analysing data from the centre of the burned and adjacent unburned areas (χ^2^  **=** 7.58, *df* = 2, *P* = 0.02), indicating that, although flowering density was higher in the burned areas across all localities, the difference was not statistically significant in one of them (Vall d’Ebo; *post hoc* analysis: *P* = 0.07; Table S6).

The effect of fire on resprouting density (number of individuals resprouting per plot) for *A. cerasiferus* also differed among localities when comparing the centre of burned areas with adjacent unburned areas (fire × locality interaction: χ^2^ = 7.15, *df* = 2, *P* = 0.03; Supplementary Data Table S6), increasing only in one of the populations (Bejís; *post hoc* analysis: estimated marginal mean = 4.68 ± 2.15, *P* < 0.001), but not in the other two localities (Table S6). No significant effect of fire on resprouting density was detected in the fire-edge population (χ^2^ = 0.51, *df* = 1, *P* = 0.47). Fire positively affected the percentage of resprouting individuals that flowered in all cases. For *Drimia maritima*, the opposite pattern was observed: fire increased resprouting density but did not affect the proportion of flowering individuals, both when comparing the centre of the burned and adjacent unburned areas and in the fire-edge population (Fig. S2, Table S4).

### Reproductive output

**Fig. 4.**
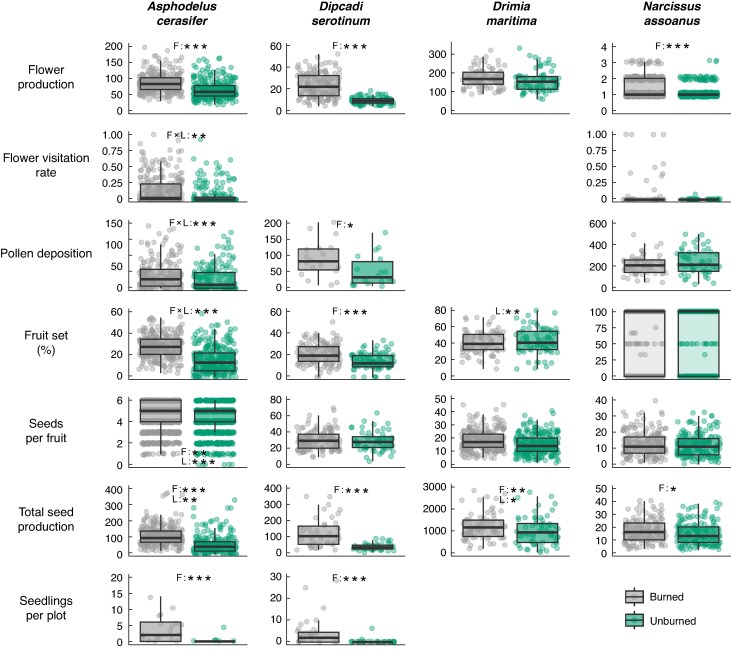
Reproductive output in unburned (green) and burned areas (grey). Each column of graphs corresponds to a species (*Asphodelus cerasifer*, *Dipcadi serotinum*, *Drimia maritima* and *Narcissus assoanus*, from left to right), and each row represents a response variable: flower production, flower visitation rate, pollen deposition (pollen grains on the stigma), fruit set (%), seeds per fruit, total seed production and seedlings per plot. Boxplot boundaries indicate the 25th and 75th percentiles, the horizontal line marks the median and whiskers extend up to 1.5 times the interquartile range. Each dot represents a sampling unit that varies depending on the response variable: an individual plant for flower production, fruit set and total seed production; a pollinator census for flower visitation rate, an individual flower for pollen deposition (i.e. a stigma); a fruit for seeds per fruit; and a plot for seedlings per plot. Note the difference in the scale between species for each response variable. Asterisks indicate the significance level of fire (F), locality (L) and their interaction (F × L) for *A. cerasifer* and *Drimia maritima*; and fire (F) only for *Dipcadi serotinum* and *N. assoanus*: *P* < 0.05 (*), *P* < 0.01 (**), *P* < 0.001 (***); non-significant comparisons are not marked. See Supplementary Data Table S7 for summary statistics.

All species showed increased reproductive output after the fire compared to the unburned areas (Fig. 4; Supplementary Data Table S7); however, there were some differences among species in the specific mechanism that enhanced reproduction.

Total flower production per individual was higher in the burned areas for all species, although not statistically significant for *Drimia maritima* (χ^2^ = 2.69, *df* = 1, *P* = 0.1). Most individuals of *N. assoanus* produced a single flower in both burned (74.2 % of individuals) and unburned areas (87.8 %), resulting in low variation in this response variable that prevented reliable statistical tests of fire effect. Nevertheless, the proportion of individuals producing multiple flowers (up to four) was significantly higher in burned areas (25.8 %) compared to unburned areas (12.2 %; chi-square test: χ^2^  **=** 27.1, *P* < 0.001), suggesting also a potential increase in flower production following fire. For *Dipcadi serotinum*, all individuals in the unburned areas produced a single scape while in the burned areas 53 % of individuals produced more than one, and up to four (Pearson’s chi-square test: χ^2^  **=** 80.9, *P* < 0.001), and each of these scapes also produced more flowers in burned areas (estimated coefficient ± s.e. = 0.47 ± 0.08, χ^2^ = 29.3, *df* = 1, *P* < 0.001).

Flower visitation rate was low for *N. assoanus* in both burned and unburned sites. In unburned areas, only a single flower visit was recorded (by a beetle from the family Nitidulidae), resulting in a flower visitation rate close to zero, whereas in burned areas, a few more were observed, mainly by *Macroglossum stellatarum* (Sphingidae), but the visitation rate remained very low (0.121 ± 0.286; mean and s.d.). Owing to the overall low number of observed pollinator visits, particularly in unburned areas, no reliable statistical analyses could be conducted to formally test the effect of fire on visitation rates. For *A. cerasifer*, flower visitation rate was higher in burned sites in two of the three localities (fire × locality interaction: χ^2^ = 11.1, *df* = 2, *P* = 0.004), but not in the third (Bejís; *post hoc* analysis: *P* = 0.65). In both burned and unburned sites across all localities, medium- to large-sized bees from the family Apidae were the main pollinators: *Apis mellifera* (accounting for 56.9 % of visits in burned vs. 23.9 % in unburned areas), *Bombus terrestris* (32.3 % vs. 29.4 %) and *Xylocopa violacea* (4.1 % vs. 22.12 %). We did not observe any pollinator visiting *Dipcadi serotinum* flowers in either burned or unburned areas; pollinator visitation rates seem to be very low and a greater number of observation minutes would probably be necessary to record any visits and differences between fire treatments. Pollen deposition on stigmas increased significantly after fire in *A. cerasifer* for two of the three localities (fire × locality interaction: χ^2^ = 38.5, *df* = 2, *P* < 0.001), but not in the third (Vall d’Ebo; *post hoc* analysis: *P* = 0.65; Supplementary Data Table S6). Fire also enhanced pollen deposition in *Dipcadi serotinum* (observed means ± s.e.: 94.7 ± 61.2 pollen grains in burned areas vs. 57.8 ± 58.2 in unburned areas; χ^2^ = 5.1, *df* = 1*, P* = 0.02), but did not differ between fire treatments for *N. assoanus* (χ^2^ = 2.86, *df* = 1*, P* = 0.09).

Fruit set was significantly higher in burned than in unburned areas for *Dipcadi serotinum* (estimated coefficient ± s.e. = 0.49 ± 0.104, χ^2^ = 21.9, *df* = 1, *P* < 0.001) and for *A. cerasifer*, with the magnitude of the increase varying among localities (fire × locality interaction: χ^2^ = 18.82, *df* = 2, *P* < 0.001; Supplementary Data Table S6). The number of seeds per fruit was also significantly higher in burned areas for *A. cerasifer* (estimated coefficient ± s.e. = 0.34 ± 0.15, χ^2^ = 5.34, *df* = 1, *P* = 0.02).

Total seed production per individual was higher in burned areas for all species (Fig. 4; Supplementary Data Table S7).

The distance to the edge of the fire was not significant in most of the models above (and thus was not included). However, there were two cases in which they showed a significant effect. In the model of seeds per fruit in *Drimia maritima*, and in the one of fruiting in *N. assoanus*, the distance was significant, and when included, the effect of fire also became significant. However, for *Drimia maritima* the fire increased the number of seeds per fruit, but it decreased with distance within the fire, while in *N. assoanus*, fire decreased fruit set but it increased with the distance from the edge (Supplementary Data Table S8). That is, these two models including the distance to the edge were inconclusive and coherent with the non-significant effect above (Table S8; Fig. 4). Overall, these results suggest that the different distances to the edge locations of the sites do not weaken our results.

The mean number of seedlings per plot was three times higher in burned than in unburned plots for *A. cerasifer* (observed means: 3.33 in burned vs. 1.33 unburned areas; χ^2^ = 14.3, *df* = 1, *P* < 0.001) and ten times higher for *Dipcadi serotinum* (3.49 vs. 0.39, χ^2^ = 39.4, *df* = 1, *P* < 0.001).

When analysing all species combined, fire had a predominantly significant positive effect on almost all stages of the reproductive cycle, as indicated by positive effect sizes (Fig. 3). In the cases of pollen deposition and seeds per fruit, the positive effect was not statistically significant (95 % confidence intervals overlapping zero) (Fig. 4; Supplementary Data Table S5). The overall mean (pooled) Hedges’ *g*, calculated across all response variables and species, was also significant and positive.

## DISCUSSION

We provide a comprehensive assessment of the reproductive consequences of fire-stimulated flowering in Mediterranean geophytes, integrating the effect of fire across multiple key stages of plant reproduction. This integrative approach covers key stages from flowering to seedling recruitment, thus capturing several of the ecological processes and demographic bottlenecks operating between reproduction and establishment. By doing so, we offer a complete understanding of the ecological and evolutionary benefits of this strategy. Fire consistently increased the reproductive output across the four geophyte species studied, with some variations across species on the specific fitness component that is significantly enhanced. This was also consistent when analysing all data combined. Although some intermediate stages (e.*g*. seed dispersal, germination or post-recruitment survival) were not measured directly, seedling recruitment under natural conditions partially reflects their combined effects. These findings support the hypothesis that fire-stimulated flowering is adaptive in fire-prone ecosystems as it confers significant reproductive benefits, via different mechanisms discussed below.

Fire increased both the density of flowering individuals and the flower production per individual in all four species. Although this is known from other geophyte species (Vickery, 2002; Diadema *et al*., 2007; Borchert and Tyler, 2009), the mechanisms underlying the stimulation of flowering in response to fire remain poorly understood. As a natural disturbance, fire reduces competition and enhances key abiotic resources, such as light and soil nutrients, by eliminating surrounding vegetation and releasing nutrients (Caon *et al*., 2014). This improvement of abiotic conditions can be the reason promoting both the initiation of flowering and the production of a greater number of flowers per individual (Coutinho, 1976; Le Maitre and Brown, 1992; Tyler and Borchert, 2003). Additionally, chemical by-products of the combustion, such as smoke, can have a role in stimulating flowering, although this has been demonstrated only in a few cases (Stone, 1951; Keeley, 1993). Leaf loss from fire could also be the cue for flowering (Taylor *et al*., 1999; Fontenele *et al*., 2020), although this is unlikely for deciduous geophytes, whose leaves are typically dry by the time fire occurs. Thus, the induction of flowering is probably driven by multiple interacting factors. In fact, for the two species where we could distinguish between resprouting and flowering individuals, *Drimia maritima* and *A. cerasifer*, the processes behind the observed increase in flowering density appear to differ. In the case of the hysteranthous *Drimia maritima*, fire appears to stimulate dormant individuals from the bulb bank, increasing the overall pool available to flower that might otherwise remain dormant for extended periods. For the synanthous *A. cerasifer*, fire does not activate the below-ground bud bank, but rather increases the flowering probability. This suggests that the mechanisms for flower stimulation are complex and vary according to each species’ life history and resource accumulation strategy (Dafni *et al*., 1981*b*).

In addition to the benefits from improved abiotic conditions, some of the studied species also experienced more favourable biotic conditions in burned areas, particularly through enhanced pollination, improving their sexual reproduction in the post-fire environment. In *A. cerasifer*, flower visitation rate was higher in burned areas in all three studied localities, although the difference was statistically significant in only two of them. This pattern was accompanied by higher pollen deposition on stigmas in burned areas of two localities, indicating that the effect of fire on pollination in *A. cerasifer* is generally positive but not uniform across localities. In all localities of *A. cerasifer*, fruit set and the number of seeds per fruit were higher in burned areas. These results align with previous work suggesting that increased flowering density and synchronization can boost pollination success in the early post-fire environment (Wagenius *et al*., 2020; Richardson and Wagenius, 2022), through both increased mate-availability within the population and the greater attraction of pollinators. Flower patches in burned areas will be reached primarily by pollinators with long flight ranges, whose relative abundance tends to increase into burned areas (Tscheulin *et al*., 2011; Ulyshen *et al*., 2024). We suggest that the higher presence of these large pollinators, often more efficient at pollinating large, open flowers such as those of *A. cerasifer*, may also have contributed to the higher pollination efficiency observed in burned areas. Previous studies on *Asphodelus ramosus* suggested that pollinator visitation and fruit set were lower in burned areas (Ne’eman *et al*., 2000), but their study was conducted 4 years after the fire, a stage at which post-fire advantages are expected to have diminished. In contrast, Potts *et al*. (2006) found that pollen deposition remained higher in burned areas even 2 years post-fire, suggesting that positive fire effects on pollination can persist beyond the immediate post-fire period, although probably at reduced intensity. Overall, while some benefits may persist beyond the immediate post-fire period, they are often diluted as the community recovers, and hence studies conducted long after the fire may underestimate the magnitude of post-fire benefits.

In contrast, *N. assoanus* showed increased flower visitation rate after fire without increasing pollen deposition, fruit set or number of seeds per fruit. This suggests that increased flower visitation by pollinators does not necessarily result in effective pollination in this species. *Narcissus assoanus* populations are highly spatially structured due to limited seed dispersal (because of the plant’s small size) and clonal reproduction (Baker *et al*., 2000*a*). In such systems, dense flowering may increase the likelihood of inbreeding, thereby reducing pollination quality despite elevated visitation rates to flowers. In *Dipcadi serotinum*, pollen deposition did increase in burned areas, but this did not translate into a higher number of seeds per fruit, again suggesting a disconnection between pollination quantity and quality. This may reflect reduced outcrossing efficiency in post-fire environments, possibly due to geitonogamy or mating among closely related individuals, as has been documented in other self-incompatible species (LoPresti *et al*., 2018).

Although fruit and seed set did not increase in all species after fire, the seed production output for the population increased in the burned areas for all species, with notable increases in both the overall number of individuals producing seeds and the total number of seeds produced per individual. This strategy of releasing large quantities of seed, coinciding with the increased nutrient availability and reduced competition after fire, is an optimal strategy for successful seedling recruitment. This expected increase in seedling recruitment was observed for *A. cerasifer* and *Dipcadi serotinum.* This close relationship between fire and recruitment suggests that, for fire-stimulated flowering species, significant recruitment events may be confined to post-fire periods with little or no recruitment occurring during the interfire interval (Keeley and Fotheringham, 2000; Keeley *et al*., 2006) as occurs in species with fire-released seed dormancy (Pausas and Lamont, 2022) and serotiny (Lamont *et al*., 2020). These recruitment events are particularly important for long-lived geophytes, as, although they are capable of clonal reproduction, sexual reproduction plays a critical role in maintaining genetic diversity essential for population resilience. Fire provides to these geophytes a window of opportunity for sexual reproduction and thus fire-stimulated flowering aligns with the best-bet evolutionary strategy typical in many fire-prone ecosystems (Pausas *et al*., 2022).

In summary, fire enhances reproductive output in Mediterranean fire-stimulated flowering geophytes at different stages of their life cycle, providing evidence of the adaptive value of this strategy where fires are recurrent. These species take advantage of post-fire conditions to maximize their reproductive success during the critical period immediately following the fire. These results contribute to the evidence that fire can play a crucial role in regulating the population dynamics of fire-adapted species beyond the typical dynamics of fire-killed seeder species. In addition, the mechanism by which fire increases sexual reproduction varies among species, underscoring the importance of species-specific traits and life history in shaping fire responses. Understanding the mechanisms behind the increase in reproductive success is essential for predicting how plant populations will respond to future fire regimes and for developing effective conservation strategies to protect fire-adapted species in fire-prone ecosystems.

## Supplementary Material

mcag048_Supplementary_Data

## Data Availability

The dataset compiled in this study is available on figshare: https://figshare.com/s/126e56288f3150de318a.

## SUPPLEMENTARY DATA

Supplementary data are available at *Annals of Botany* online and consist of the following. Figure S1. Localities and sampling sites of the study. Figure S2. Number of individuals resprouting per plot in the burned and unburned areas for *Asphodelus cerasifer* and *Drimia maritima.* Table S1. Results of the preliminary pollinator exclusion experiment in *Dipcadi serotinum.* Table S2. Information on wildfires for each sampled locality. Table S3. Summary of the structure of the fitted models for each species. Table S4. Summary statistics for effect of fire on the resprouting and flowering density of the study species. Table S5. Fire effect sizes for each stage of the reproductive cycle across species. Table S6. *Post hoc* pairwise comparisons for the significant fire × locality interactions in *Asphodelus cerasifer*. Table S7. Summary statistics for the effect of fire on the reproductive output of the study species. Table S8. Summary statistics for effects of distance to the edge and fire on reproductive output of the study species.

## AI ASSISTANCE

No AI tools were used for data analysis, interpretation or the generation of scientific content in this article. AI-assisted tools were used for minor language editing.
