# Supplementary material for: Fire-stimulated flowering enhances multiple plant fitness components
Source: Ann Bot. 2026 Mar 16;137(7):2198–208. doi: 10.1093/aob/mcag048 (PMC13319519; doi:10.1093/aob/mcag048)
Supplement: mcag048_Supplementary_Data [file mcag048_supplementary_data.docx]

**Fire-stimulated flowering enhances multiple plant fitness components**

**SUPPORTING INFORMATION**

FIGURE S1. Localities and sampling sites of the study.

FIGURE S2. Number of individuals resprouting per plot in the burned and unburned areas for *Asphodelus cerasifer* and *Drimia maritima.*

TABLE S1. Results of the preliminary pollinator exclusion experiment in *Dipcadi serotinum.*

TABLE S2. Information on wildfires for each sampled locality.

TABLE S3. Summary of the structure of the fitted models for each species.

TABLE S4. Summary statistics for effect of fire on the resprouting and flowering density of the study species.

TABLE S5. Fire effect sizes for each stage of the reproductive cycle across species.

TABLE S6. Post-hoc pairwise comparisons for the significant fire × locality interactions in *Asphodelus cerasifer*.

TABLE S7. Summary statistics for effect of fire on the reproductive output of the study species.

TABLE S8. Summary statistics for effects of distance to the edge and fire on reproductive output of the study species.

**
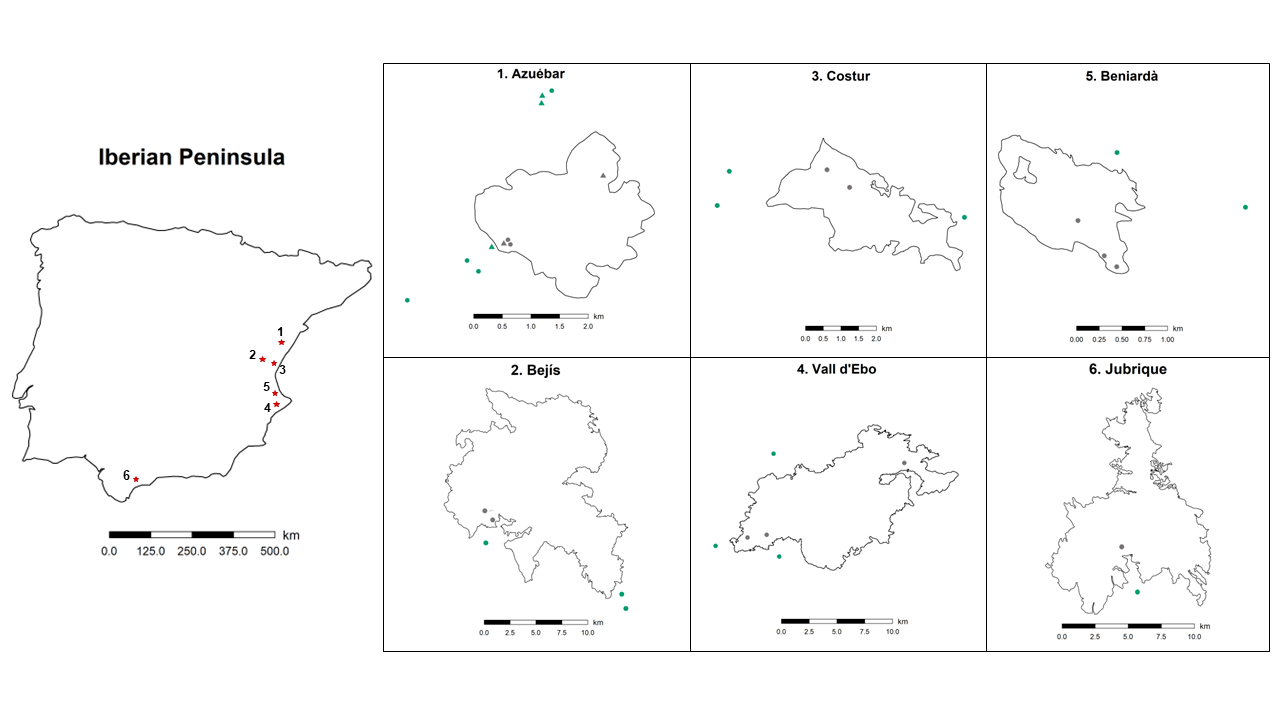
FIGURE S1.** Location of the six study localities within the Iberian Peninsula. Right panels show the fire wildfire perimeters and sampling sites within each locality. Within each locality, sampling sites in burned areas are shown in grey symbols, and sites in unburned areas in green symbols. In Azuébar, dots represent sites sampled for *Asphodelus cerasifer* and triangles represent sites sampled for *Dipcadi serotinum. Asphodelus cerasifer* was sampled also sampled in Bejís and Vall d’Ebo; *Narcissus assoanus* in Costur; and *Drimia maritima* in Beniardà and Jubrique.

**FIGURE S2.** Number of individuals resprouting per plot in the burned (grey) and unburned (green) areas for *Asphodelus cerasifer* and *Drimia maritima.* The lower and upper boundaries of the boxplot indicate the 25th and 75th percentiles, the horizontal line within the box marks the median and the whiskers indicate data range. Shaded portion of each graph shows data from the partially burned populations in the edge of burned areas and non-shaded portion, data from the sites in the center of the burned areas and adjacent unburned areas. Asterisks indicate significance levels: p < 0.05 (*), p < 0.01 (**), p < 0.001 (***), p > 0.5 (n.s.). See Table S4 for summary statistics.

**
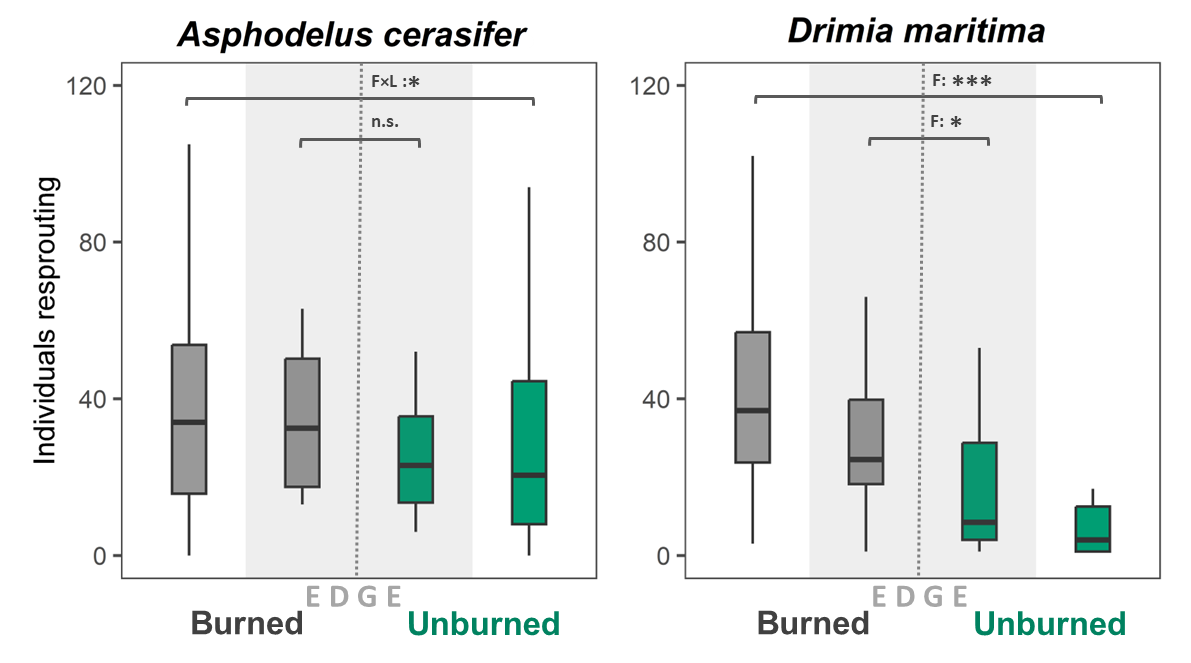
**

**TABLE S1**. Results of the preliminary pollinator exclusion experiment to test the dependence of *D. serotinum* on pollinators for fruit production (*N* = 11 plants). Plants were collected from natural populations and maintained under greenhouse conditions. Of the 11 individuals selected, 4 had their floral scapes bagged with fine mesh to prevent pollinating insect visitors (pollinator exclusion = yes), while the remaining 7 were left unbagged to allow open pollination (pollinator exclusion = no). The number of flowers and fruits was recorded for each plant, and fruit set was calculated as the percentage of flowers that developed into fruits.

| **Plant code** | **Pollinator exclusion** | **Flowers** | **Fruits** | **Fruit set (%)** |
| --- | --- | --- | --- | --- |
| AZ1.11 | yes | 13 | 0 | 0 |
| AZ1.34 | yes | 21 | 0 | 0 |
| AZ2.13 | yes | 10 | 0 | 0 |
| AZ2.30 | yes | 7 | 0 | 0 |
| AZ1.24 | no | 7 | 4 | 57.1 |
| AZ1.50 | no | 10 | 3 | 30.0 |
| AZ1.64 | no | 8 | 1 | 12.5 |
| AZ2.32 | no | 18 | 0 | 0 |
| AZ2.34 | no | 13 | 4 | 30.8 |
| AZ2.36 | no | 15 | 4 | 26.3 |
| AZ2.43 | no | 19 | 1 | 5.3 |

**TABLE S2.** Information on wildfires for each sampled locality (burned areas), including area (in hectares) and geographical coordinates indicating the center of the burned area.

| **Species** | **Locality** | **Wildfire area**  **(ha)** | **Wildfire coordinates** |
| --- | --- | --- | --- |
| *Asphodelus cerasifer* | Azuébar  (Castellón) | 420 | 39.84397, -0.38758 |
|  | Bejís  (Castellón) | 19159 | 39.89162, -0.70381 |
|  | Vall d’Ebo  (Alicante) | 12151 | 38.78816, -0.20886 |
| *Drimia maritima* | Beniardà  (Alicante) | 134 | 38.70574, -0.22165 |
|  | Jubrique  (Málaga) | 9731 | 36.53417, - 5.16397 |
| *Dipcadi serotinum* | Azuébar  (Castellón) | 420 | 39.84397, -0.38758 |
| *Narcissus assoanus* | Costur  (Castellón) | 818 | 40.15271, -0.19268 |

**TABLE S3.** Summary of structure of the fitted models for each species. For each response variable, the table indicates the model type (GLMM: Generalized Linear Mixed Models or LMM: Linear Mixed Models), error distribution, and fixed and random effects structure.

| **Response variable** | **Model type** | **Error**  **distribution** | **Model structure** | |
| --- | --- | --- | --- | --- |
| ***Asphodelus cerasifer*** | | | | |
| Resprouting density | GLMM | Poisson | Center: ~Fire*Locality + (1\|Site)  Edge: Fire + (1\|Site) | |
| Flowering density | GLMM | Poisson | Center: ~Fire*Locality + (1\|Site)  Edge: ~Fire + (1\|Site) | |
| Flowering proportion | GLMM | Binomial | Center: ~Fire*Localiy + Fire | |
| Flower production (log) | LMM | Normal | ~Fire*Locality + (1\|Site) | |
| Flower visitation rate | GLMM | Binomial | ~Fire*Locality + (1\|Site) | |
| Pollen deposition | GLMM | Negative binomial | ~Fire*Locality + (1\|Site) | |
| Fruit set | GLMM | Binomial | ~Fire*Locality + (1\|Site) + (1\|Plant ID) | |
| Seeds per fruit (square-root) | LMM | Normal | ~Fire*Locality + (1\|Site) + (1\|Plant ID) | |
| Total seed production (square-root) | LMM | Normal | ~Fire*Locality + (1\|Site) | |
| Seedlings per plot | GLMM | Negative binomial | ~Fire*Locality + (1\|Site) | |
| ***Drimia maritima*** | | | |  |
| Resprouting density | GLMM | Poisson | ~Fire + (1\|Site) | |
| Flowering density | GLMM | Poisson | ~Fire + (1\|Site) | |
| Flowering proportion | GLMM | Binomial | ~Fire + (1\|Site) | |
| Flower production (log) | LMM | Normal | ~Fire*Locality + (1\|Site) | |
| Fruit set | GLMM | Binomial | ~Fire*Locality + (1\|Site) + (1\|Plant ID) | |
| Seeds per fruit (square-root) | LMM | Normal | ~Fire*Locality + (1\|Site) + (1\|Plant ID) | |
| Total seed production (square-root) | LMM | Normal | ~Fire*Locality + (1\|Site) | |
| ***Dipcadi serotinum*** | | | |  |
| Flowering density | GLMM | Poisson | ~Fire + (1\|Site) | |
| Flower production (log) | LMM | Normal | ~Fire + (1\|Site) | |
| Pollen deposition (log) | LMM | Normal | ~Fire + (1\|Site) | |
| Fruit set | GLMM | Binomial | ~Fire + (1\|Site) + (1\|Plant ID) | |
| Seeds per fruit (square-root) | LMM | Normal | ~Fire + (1\|Site) + (1\|Plant ID) | |
| Total seed production (square-root) | LMM | Normal | ~Fire + (1\|Site) | |
| Seedlings per plot | GLMM | Negative binomial | ~Fire + (1\|Site) | |
| ***Narcissus assoanus*** | | | |  |
| Flowering density | GLMM | Poisson | ~Fire + (1\|Site) | |
| Flower production (log) | LMM | Normal | ~Fire + (1\|Site) | |
| Pollen deposition | LMM | Normal | ~Fire + (1\|Site) | |
| Fruit set | GLMM | Binomial | ~Fire + (1\|Site) + (1\|Plant ID) | |
| Seeds per fruit (square-root) | LMM | Normal | ~Fire + (1\|Site) + (1\|Plant ID) | |
| Total seed production (square-root) | LMM | Normal | ~Fire + (1\|Site) | |

**TABLE S4**. Summary statistics for effect of fire, locality and their interaction, on the resprouting and flowering density and on the proportion of individuals flowering of *Asphodelus cerasifer*, *Drimia maritima*; and for effect of fire on flowering density for *Dipcadi serotinum* and *Narcissus assoanus. N* indicates sample sizes per model (number of plots). Model statistics: parameter estimate **±** standard error (SE), *t/z* value (from LMM/GLMM), degrees of freedom (*df*), χ2 from Wald chi-squared test, and *p* value for significance level.

|  |  |  | ***Asphodelus cerasifer*** | | | | | |
| --- | --- | --- | --- | --- | --- | --- | --- | --- |
|  |  | ***N*** | **Predictor** | **Estimate ± SE** | ***z* value** | ***df*** | **χ^2^** | ***p val*ue** |
| **Center** | Resprouting | 205 | Fire (B) | 0.888 ± 0.504 | 1.765 | 1 | 3.114 | 0.776 |
|  |  |  | Locality (Bj) | 0.533 ± 0.440 | 1.212 | 2 | 4.635 | 0.098 |
|  |  |  | Locality (V) | 1.066 ± 0.496 | 2.151 |  |  |  |
|  |  |  | Fire × Locality (B×Bj) | 0.654 ± 0.682 | 0.959 | 2 | 7.152 | **0.028** |
|  |  |  | Fire × Locality (B×V) | -1.093 ± 0.689 | -1.587 |  |  |  |
|  | Flowering | 205 | Fire (B) | 2.656 ± 0.512 | 5.189 | 1 | 26.925 | **<0.001** |
|  |  |  | Locality (Bj) | 2.399 ± 0.460 | 5.218 | 2 | 29.304 | **<0.001** |
|  |  |  | Locality (V) | 2.193 ± 0.511 | 4.290 |  |  |  |
|  |  |  | Fire × Locality (B×Bj) | -0.835 ± 0.671 | -1.244 | 2 | 7.577 | **0.023** |
|  |  |  | Fire × Locality (B×V) | -1.855 ± 0.681 | -2.724 |  |  |  |
|  | Proportion | 205 | Fire (B) | 2.326 ± 0.562 | 4.138 | 1 | 26.882 | **<0.001** |
|  |  |  | Locality (Bj) | 2.472 ± 0.493 | 5.018 | 2 | 24.529 | **<0.001** |
|  |  |  | Locality (V) | 1.446 ± 0.564 | 2.566 |  |  |  |
|  |  |  | Fire × Locality (B×Bj) | -1.534 ± 0.744 | -2.062 | 2 | 4.472 | 0.107 |
|  |  |  | Fire × Locality (B×V) | -0.576 ± 0.762 | -0.756 |  |  |  |
| **Edge** | Resprouting | 8 | Fire (B) | 0.359 ± 0.502 | 0.716 | 1 | 0.512 | 0.47 |
|  | Flowering | 8 | Fire (B) | 1.600 ± 0.605 | 2.644 | 1 | 6.990 | **0.0082** |
|  | Proportion | 8 | Fire (B) | 1.597 ± 0.372 | 4.296 | 1 | 18.458 | **< 0.001** |
|  |  | ***Drimia maritima*** | | | | | | |
|  |  | ***N*** | **Predictor** | **Estimate ± SE** | ***z* value** | ***df*** | **χ^2^** | ***p val*ue** |
| **Center** | Resprouting | 95 | Fire (B) | 1.932 ± 0.385 | 5.023 | 1 | 25.227 | **< 0.001** |
|  | Flowering | 95 | Fire (B) | 1.487 ± 0.579 | 2.570 | 1 | 6.606 | **0.010** |
|  | Proportion | 95 | Fire (B) | -0.493 ± 0.493 | -0.999 | 1 | 0.999 | 0.32 |
| **Edge** | Resprouting | 28 | Fire (B) | 0.855 ± 0.369 | 2.318 | 1 | 5.375 | **0.020** |
|  | Flowering | 28 | Fire (B) | 0.693 ± 0.218 | 3.173 | 1 | 10.07 | **0.0015** |
|  | Proportion | 28 | Fire (B) | -0.023 ± 0.249 | -0.092 | 1 | 0.0085 | 0.93 |

|  |  | ***Dipcadi serotinum*** | | | | | | |
| --- | --- | --- | --- | --- | --- | --- | --- | --- |
|  |  | ***N*** | **Predictor** | **Estimate ± SE** | ***z* value** | ***df*** | **χ^2^** | ***p val*ue** |
| **Center** | Flowering | 115 | Fire (B) | 0.110 ± 0.110 | 15.219 | 1 | 231.61 | **< 0.001** |
| **Edge** | Flowering | 8 | Fire (B) | 0.600 ± 0.600 | 4.726 | 1 | 22.339 | **< 0.001** |
|  | | | | | | | | |
|  |  | ***Narcissus assonaus*** | | | | | | |
|  |  | ***N*** | **Predictor** | **Estimate ± SE** | ***z* value** | ***df*** | **χ^2^** | ***p val*ue** |
| **Center** | Flowering | 129 | Fire (B) | 1.326 ± 0.306 | 4.331 | 1 | 18.760 | **< 0.001** |
| **Edge** | Flowering | 30 | Fire (B) | 4.435 ± 1.843 | 1.843 | 1 | 5.792 | **0.016** |

**TABLE S5**. Model-estimated mean (pooled) fire effect sizes (Hedges’ g) and 95% confidence intervals for each stage of the reproductive cycle across species and overall effect across stages and species (see also Fig. 4 in the main text). Positive effect sizes indicate a positive effect of fire, whereas negative values indicate a negative effect of fire. Effects were considered statistically significant when 95% confidence intervals did not include zero.

|  | **Fire effect size** | **ci.lb** |
| --- | --- | --- |
| Flowering density | 0.60645 | (0.170, 1.043) |
| Flower production | 0.67702 | (0.457, 0.897) |
| Flower visitation rate | 0.63497 | (0.360, 0.909) |
| Pollen deposition | 0.15092 | (-0.119, 0.420) |
| Fruit set | 0.55747 | (0.338, 0.77) |
| Seeds per fruit | 0.18393 | (-0.027, 0.395) |
| Total seed production | 0.63851 | (0.399, 0.878) |
| Seedlings per plot | 0.60645 | (0.170, 1.043) |
| Overall | 0.4971 | (0.293, 0.701) |

**TABLE S6**. Post-hoc pairwise comparisons for the significant fire × locality interactions in *Asphodelus cerasifer*. The table shows estimated marginal means (EMMs) ± **±** standard error (SE) with lower and upper 95% confidence limits (LCL–UCL) for each fire × locality combination. For each locality, estimated ratios **±** standard error (SE) represent the ratio between estimated marginal means of burned and unburned treatments on the response scale, and *z* ratios and *p* values for significance level for each contrast.

|  | **Locality** | **Fire** | **EMMs ± SE** | **LCL - UCL** | **Estimated**  **ratio** **± SE** | ***z* ratio** | ***p* value** |
| --- | --- | --- | --- | --- | --- | --- | --- |
| Resprouting density | Azuébar | Unburned | 9.4 ± 3.19 | 4.83 - 18.3 | 2.432 ± 1.225 | 1.765 | 0.078 |
|  |  | Burned | 22.9 ± 8.48 | 11.05 - 47.3 |  |  |  |
|  | Bejís | Unburned | 16.0 ± 4.49 | 9.24 - 27.7 | 4.676 ± 2.152 | 3.351 | **<0.001** |
|  |  | Burned | 74.8 ± 27.31 | 36.61 - 153.0 |  |  |  |
|  | Vall d’Ebo | Unburned | 27.3 ± 9.85 | 13.45 - 55.4 | 0.815 ± 0.384 | -0.434 | 0.664 |
|  |  | Burned | 22.2 ± 6.71 | 12.31 - 40.2 |  |  |  |
| Flowering density | Azuébar | Unburned | 0.747 ± 0.281 | 0.358 - 1.56 | 14.24 ± 7.292 | 5.189 | **<0.001** |
|  |  | Burned | 10.647 ± 3.739 | 5.349 - 21.19 |  |  |  |
|  | Bejís | Unburned | 8.228 ± 2.183 | 4.891 - 13.84 | 6.18 ± 2.681 | 4.201 | **<0.001** |
|  |  | Burned | 50.861 ± 17.445 | 25.967 - 99.62 |  |  |  |
|  | Vall d’Ebo | Unburned | 6.700 ± 2.323 | 3.396 - 13.22 | 2.23 ± 0.999 | 1.788 | 0.074 |
|  |  | Burned | 14.937 ± 4.247 | 8.555 - 26.08 |  |  |  |
| Flower visitation rate | Azuébar | Unburned | 0.0007 ± 0.0009 | 5.62×10^-5^ - 0.009 | 40.73 ± 54.73 | 2.759 | **0.006** |
|  |  | Burned | 0.029 ± 0.010 | 1.45×10^-2^ - 0.057 |  |  |  |
|  | Bejís | Unburned | 0.016 ± 0.007 | 6.84×10^-3^ - 0.036 | 1.28 ± 0.703 | 0.453 | 0.650 |
|  |  | Burned | 0.020 ± 0.008 | 9.27×10^-3^ - 0.043 |  |  |  |
|  | Vall d’Ebo | Unburned | 0.004 ± 0.001 | 1.94×10^-3^ - 0.009 | 10.32 ± 4.848 | 4.968 | **<0.001** |
|  |  | Burned | 0.041 ± 0.013 | 2.11×10^-2^ - 0.077 |  |  |  |
| Pollen deposition | Azuébar | Unburned | 0.551 ± 0.168 | 0.303 - 1.0 | 21.721 ± 9.023 | 7.410 | **<0.001** |
|  |  | Burned | 11.962 ± 3.360 | 6.897 - 20.7 |  |  |  |
|  | Bejís | Unburned | 19.275 ± 4.904 | 11.706 - 31.7 | 2.004 ± 0.675 | 2.065 | **0.039** |
|  |  | Burned | 38.633 ± 8.883 | 24.618 - 60.6 |  |  |  |
|  | Vall d’Ebo | Unburned | 43.398 ± 11.221 | 26.144 - 72.0 | 0.764 ± 0.273 | -0.754 | 0.451 |
|  |  | Burned | 33.141 ± 8.242 | 20.356 - 54.0 |  |  |  |
| Fruit set | Azuébar | Unburned | 0.045 ± 0.006 | 0.034 - 0.060 | 5.31 ± 1.154 | 7.693 | **<0.001** |
|  |  | Burned | 0.202 ± 0.025 | 0.157 - 0.256 |  |  |  |
|  | Bejís | Unburned | 0.183 ± 0.019 | 0.148 - 0.224 | 1.72 ± 0.338 | 2.776 | **0.005** |
|  |  | Burned | 0.278 ± 0.030 | 0.223 - 0.341 |  |  |  |
|  | Vall d’Ebo | Unburned | 0.185 ± 0.022 | 0.146 - 0.232 | 1.77 ± 0.336 | 3.029 | **0.002** |
|  |  | Burned | 0.287 ± 0.025 | 0.241 - 0.339 |  |  |  |

**TABLE S7**. Summary statistics for effects of fire, locality, and their interaction on the reproductive output of *Asphodelus cerasifer* and *Drimia maritima*, and for the effect of fire only on *Dipcadi serotinum* and *Narcissus assoanus*. *N* indicates sample sizes per model. Sampling units differ by response variable: individual plants (flower production, fruit set, total seed production), pollinator censuses (flower visitation rate), flowers (pollen deposition), fruits (seeds per fruit), and plots (seedlings per plot).

Model statistics: parameter estimate **±** standard error (SE), *t/z* value (from LMM/GLMM), degrees of freedom (df), χ2 from Wald chi-squared test, and *p* value for significance level.

|  | ***Asphodelus cerasifer*** | | | | | | |
| --- | --- | --- | --- | --- | --- | --- | --- |
|  | ***N*** | **Predictor** | **Estimate ± SE** | ***t/z* value** | **df** | **χ^2^** | ***p* value** |
| Flower visitation  rate | 496 | Fire (B) | 3.707 ± 1.344 | 2.759 | 1 | 7.612 | **0.006** |
|  |  | Locality (Bj) | 3.084 ± 1.358 | 2.272 | 2 | 10.069 | **0.006** |
|  |  | Locality (V) | 1.724 ± 1.340 | 1.286 |  |  |  |
|  |  | Fire × Locality (B×Bj) | -3.458 ± 1.499 | -2.386 | 2 | 11.099 | **0.004** |
|  |  | Fire × Locality (B×V) | -1.373 ± 1.421 | -0.966 |  |  |  |
| Pollen  deposition | 357 | Fire (B) | 3.078 ± 0.415 | 7.410 | 1 | 54.907 | **<0.001** |
|  |  | Locality (Bj) | 3.555 ± 0.402 | 8.853 | 2 | 127.004 | **<0.001** |
|  |  | Locality (V) | 4.367 ± 0.401 | 10.896 |  |  |  |
|  |  | Fire × Locality (B×Bj) | -2.383 ± 0.539 | -4.419 | 2 | 38.460 | **<0.001** |
|  |  | Fire × Locality (B×V) | -3.348 ± 0.547 | -6.117 |  |  |  |
| Flower  production | 563 | Fire (B) | 0.486 ± 0.142 | 3.425 | 1 | 12.047 | **<0.001** |
|  |  | Locality (Bj) | 0.115 ± 0.126 | 0.906 | 2 | 0.601 | 0.740 |
|  |  | Locality (V) | 0.268 ± 0.135 | 1.984 |  |  |  |
|  |  | Fire × Locality (B×Bj) | -0.212 ± 0.196 | -1.080 | 2 | 4.252 | 0.119 |
|  |  | Fire × Locality (B×V) | -0.397 ± 0.193 | -2.062 |  |  |  |
| Fruit set | 563 | Fire (B) | 1.670 ± 0.217 | 7.693 | 1 | 59.181 | **<0.001** |
|  |  | Locality (Bj) | 1.542 ± 0.196 | 7.859 | 2 | 76.020 | **<0.001** |
|  |  | Locality (V) | 1.560 ± 0.208 | 7.488 |  |  |  |
|  |  | Fire × Locality (B×Bj) | -1.126 ± 0.292 | -3.849 | 2 | 18.816 | **<0.001** |
|  |  | Fire × Locality (B×V) | -1.097 ± 0.288 | -3.809 |  |  |  |
| Seeds  per fruit | 2338 | Fire (B) | 0.337 ± 0.149 | 2.256 | 1 | 5.345 | **0.021** |
|  |  | Locality (Bj) | 0.473 ± 0.137 | 3.459 | 2 | 19.336 | **<0.001** |
|  |  | Locality (V) | 0.507 ± 0.132 | 3.854 |  |  |  |
|  |  | Fire × Locality (B×Bj) | -0.236 ± 0.201 | -1.177 | 2 | 1.796 | 0.407 |
|  |  | Fire × Locality (B×V) | -0.220 ± 0.178 | -1.237 |  |  |  |
| Seed production | 456 | Fire (B) | 6.532 ± 1.921 | 3.400 | 1 | 19.233 | **<0.001** |
|  |  | Locality (Bj) | 4.934 ± 1.827 | 2.701 | 2 | 8.500 | **0.014** |
|  |  | Locality (V) | 5.684 ± 1.871 | 3.038 |  |  |  |
|  |  | Fire × Locality (B×Bj) | -3.519 ± 2.296 | -1.533 | 2 | 3.110 | 0.2112 |
|  |  | Fire × Locality (B×V) | -3.852 ± 2.262 | -1.703 |  |  |  |
| Seedlings per plot | 40 | Fire (B) | 2.657 ± 0.701 | 3.787 | 1 | 14.345 | **<0.001** |

**TABLE S7.** (continued)

|  | ***Drimia maritima*** | | | | | | |
| --- | --- | --- | --- | --- | --- | --- | --- |
|  | ***N*** | **Predictor** | **Estimate ± SE** | ***t/z* value** | **df** | **χ^2^** | ***p* value** |
| Flower  production | 160 | Fire (B) | 0.030 ± 0.079 | 0.383 | 1 | 2.691 | 0.101 |
|  |  | Locality (J) | -0.205 ± 0.104 | -1.977 | 1 | 0.964 | 0.326 |
|  |  | Fire × Locality (B×J) | 0.259 ± 0.144 | 1.799 | 1 | 3.237 | 0.072 |
| Fruit set | 160 | Fire (B) | -0.134 ± 0.119 | -1.127 | 1 | 0.434 | 0.487 |
|  |  | Locality (J) | -0.454 ± 0.166 | -2.728 | 1 | 8.455 | **0.004** |
|  |  | Fire × Locality (B×J) | 0.232 ± 0.227 | 1.022 | 1 | 1.044 | 0.307 |
| Seeds  per fruit | 535 | Fire (B) | 0.357 ± 0.253 | 1.407 | 1 | 3.477 | 0.062 |
|  |  | Locality (B) | -0.233 ± 0.305 | -0.765 | 1 | 0.854 | 0.355 |
|  |  | Fire × Locality (B×J) | 0.070 ± 0.429 | 0.163 | 1 | 0.026 | 0.871 |
| Seed production | 141 | Fire (B) | 1.944 ± 1.934 | 1.031 | 1 | 5.012 | **0.025** |
|  |  | Locality (B) | -7.269 ± 2.591 | -2.805 | 1 | 5.489 | **0.019** |
|  |  | Fire × Locality (B×J) | 5.929 ± 3.460 | 1.629 | 1 | 2.653 | 0.103 |
|  | | | | | | | |
|  | ***Dipcadi serotinum*** | | | | | | |
|  | ***N*** | **Predictor** | **Estimate ± SE** | ***t/z* value** | **df** | **χ^2^** | ***p* value** |
| Flower production | 251 | Fire (B) | 0.836 ± 0.142 | 5.901 | 1 | 34.826 | **<0.001** |
| Pollen deposition | 38 | Fire (B) | 0.774 ± 0.343 | 2.258 | 1 | 5.099 | **0.024** |
| Fruit set | 251 | Fire (B) | 0.489 ± 0.104 | 4.682 | 1 | 21.918 | **<0.001** |
| Seeds per fruit | 182 | Fire (B) | 0.270 ± 0.311 | 0.868 | 1 | 0.753 | 0.385 |
| Seed production | 87 | Fire (B) | 5.082 ± 0.745 | 6.821 | 1 | 46.529 | **<0.001** |
| Seedlings per plot | 84 | Fire (B) | 3.388 ± 0.539 | 6.279 | 1 | 39.425 | **<0.001** |
|  | | | | | | | |
|  | ***Narcissus assoanus*** | | | | | | |
|  | ***N*** | **Predictor** | **Estimate ± SE** | ***t/z* value** | **df** | **χ^2^** | ***p* value** |
| Pollen deposition | 112 | Fire (B) | - 32.50 **±** 19.22 | -1.692 | 1 | 2.863 | 0.091 |
| Fruit set | 932 | Fire (B) | 0.343 **±** 0.674 | 0.509 | 1 | 0.259 | 0.61 |
| Seeds per fruit | 367 | Fire (B) | 0.127 **±** 0.121 | 1.045 | 1 | 1.092 | 0.29 |
| Seed production | 288 | Fire (B) | 0.352 **±** 0.141 | 2.496 | 1 | 6.228 | **0.013** |

**TABLE S8.** Summary statistics for effects of distance to the edge and fire (locality and their interaction, where applicable) on pollination-related response variables. Results are presented only for models where distance to the edge was retained as a significant predictor.

Model statistics: parameter estimate **±** standard error (SE), *t/z* value (from LMM/GLMM), degrees of freedom (df), χ2 from Wald chi-squared test, and *p* value for significance level.

|  | ***Drimia maritima*** | | | | | | |
| --- | --- | --- | --- | --- | --- | --- | --- |
|  | ***N*** | **Predictor** | **Estimate ± SE** | ***z* value** | **df** | **χ^2^** | ***p* value** |
| Seeds per fruit | 141 | Fire (B) | 0.767 ± 0.164 | 4.671 | 1 | 15.354 | **<0.001** |
|  |  | Locality (J) | -0.180 ± 0.114 | -1.587 | 1 | 0.159 | 0.690 |
|  |  | Fire × Locality (B×J) | 0.905 ± 0.290 | 3.117 | 1 | 9.718 | 0.002 |
|  |  | Distance to the edge | -0.524 ± 0.146 | -3.582 | 1 | 12.828 | **<0.001** |
|  |  |  |  |  |  |  |  |
|  |  |  |  |  |  |  |  |
|  | ***Naricssus assoanus*** | | | | | | |
|  | ***N*** | **Predictor** | **Estimate ± SE** | ***z* value** | **df** | **χ^2^** | ***p* value** |
| Fruit set | 535 | Fire (B) | -7.204 ± 1.446 | -4.984 | 1 | 24.836 | **<0.001** |
|  |  | Distance to the edge | 3.825 ± 0.722 | 5.294 | 1 | 28.030 | **<0.001** |
